# Reversal of multidrug resistance by the anti-malaria drug artesunate in the esophageal cancer Eca109/ABCG2 cell line

**DOI:** 10.3892/ol.2013.1545

**Published:** 2013-08-23

**Authors:** LIANG LIU, LIAN FU ZUO, JIAN WEN GUO

**Affiliations:** Department of Flow Cytometry Analysis, Tumor Institute, The Fourth Hospital of Hebei Medical University, Shijiazhuang, Hebei 050011, P.R. China

**Keywords:** chemotherapy, esophageal cancer, multidrug resistance

## Abstract

The overexpression of ATP-binding cassette (ABC) transporters confers multidrug resistance (MDR) to tumor cells. ABCG2 is a member of the ABC superfamily. The present study aimed to investigate the correlation between ABCG2 expression and the MDR of esophageal cancer and to estimate the therapeutic benefit of downregulating ABCG2 expression and reversing chemoresistance in esophageal cells using artesunate (Art). The Eca109/ABCG2 cell line was established by transfecting the ABCG2 gene into Eca109 cells. The Eca109/ABCG2 esophageal cancer cells with ABCG2 gene overexpression were resistant to adriamycin (ADM), daunorubicin (DNR) and mitoxantrone (MIT), which indicated that ABCG2 may be associated with drug resistance in esophageal cancer. Art is a noteworthy antimalarial agent, particularly in severe and drug-resistant cancer cases, as Art is able to reverse drug resistance. In the present study, Art also exerted profound anticancer activity. The mechanism for the reversal of multidrug resistance by Art in esophageal carcinoma was analyzed using cellular experiments, but still remains largely unknown.

## Introduction

Esophageal carcinoma is one of the most common malignant gastrointestinal tumors (1). The incidence of esophageal carcinoma is high in China and it is a great threat to human health. Chemotherapy is a combined modality therapy that is used in the treatment of esophageal carcinoma, however, multidrug resistance (MDR) is a major barrier to the success of chemotherapy, particularly in recurrent tumors. MDR is the resistance of cancer cells to multiple classes of chemotherapeutic drugs that may be structurally and mechanistically unrelated. When tumor cells develop drug resistance, they become resistant not only to the treated drug, but also to a variety of structurally and functionally unrelated drugs. Since the therapeutic effect is determined by this resistance, MDR has become a focus for research. There is accumulating evidence that the active export of anticancer drugs from cells is one of the major mechanisms of MDR (2,3). Tumor cells often gain drug resistance through the overexpression of membrane transport proteins that effectively efflux anticancer drugs (4,5). It has been convincingly documented that several ATP-dependent drug transporters may cause drug resistance in cancer cells by actively extruding clinically administered chemotherapeutic drugs (6). The increased transmembrane efflux of antitumor drugs is one of the best-characterized mechanisms of MDR and is mediated through the overexpression of ATP-binding cassette (ABC) transporter superfamily members. The well-known major drug transporters, ABCB1 (P-glycoprotein or MDR1) and ABCG2 (BCRP/MXR/ABCP), have been characterized in detail with respect to their structures and functions (7). These drug transporters belong to the human ABC transporter gene family that consists of at least 48 gene members. The overexpression of ABCG2 has been reported to confer drug resistance upon malignant cells to various chemotherapeutic drugs (8,9).

Artesunate (Art) is a remarkable antimalarial agent, particularly in severe and drug-resistant cases (10–12). Art has also been demonstrated to exert profound anticancer activity (13–15). Art, a powerful antimalarial herbal compound, has been shown to inhibit the growth of various tumor cell lines *in vitro* and xenografted carcinoma in mice *in vivo*. Art has also been shown to inhibit the growth of esophageal cancer cells. Art may have anticancer effects on drug-resistant cells, indicating that the compound may reverse the drug resistance of cancer cells (16–18). Art has few adverse effects, so it may be developed into a drug to reverse MDR.

In the present study, the gene and protein expression of ABCG2 was detected by various experimental methods, to study the correlation between ABCG2 expression and the resistance of esophageal carcinoma. An Eca109/ABCG2 MDR cell was established by transfecting the ABCG2 gene into Eca109 cells. The ABCG2 expression level and drug efflux of the Eca109/ABCG2 cells was assessed using RT-PCR, western blot analysis and flow cytometry.

The mechanism for the reversal of multidrug resistance by Art in esophageal carcinoma was analyzed using cellular experiments. The correlation between ABCG2 expression in esophageal carcinoma and MDR, and the reversal of MDR by Art were investigated in the present study. These results may be beneficial to the chemotherapy of esophageal carcinoma in the clinic.

## Materials and methods

### Chemicals and reagents

Geneticin (G418), dimethyl sulfoxide (DMSO), trypsin, RPMI-1640 and a 3-[4,5-dimethylthiazol-2-yl]-2,5-diphenyltetrazolium bromide (MTT) kit were purchased from Sigma-Aldrich (St. Louis, MO, USA). The Lipofectamine™ 2000 kit was purchased from Invitrogen (Carlsbad, CA, USA). Fluorescein isothiocyanate (FITC)-conjugated ABCG2 antibodies were purchased from Biolegend (San Diego, CA, USA).

### ABCG2 gene transfection

PCDNA3.1-ABCG2 plasmids containing ABCG2 cDNA were purchased from Jing Sai Co. (Wuhan, China). Lipofectamine 2000 (Invitrogen) was used as a transfection reagent, according to the manufacturer’s instructions, and positive cell clones were selected using 600 mg/l G418 subsequent to being transfected for 72 h. The Eca109 cells that were transfected with PCDNA3.1 served as the control group. The Eca109 cells that were transfected with PCDNA3.1-ABCG2 and PCDNA3.1 were termed the Eca109/ABCG2 and Eca109/PCDNA3.1 cells, respectively. To ascertain the efficacy and specificity of the transfection, ABCG2 mRNA and protein levels were monitored using RT-PCR, western blot analysis and flow cytometry, respectively.

### Cells and cell culture

The Eca109 esophageal cancer cell line was obtained from the Tumor Institute of the Fourth Hospital of Hebei Medical University (Shijiazhuang, China). The Eca109 cells were maintained in RPMI-1640 supplemented with 10% fetal bovine serum (FBS), 5% penicillin (100 U/ml) and streptomycin (100 mg/ml) in a humidified atmosphere of 95% air and 5% CO_2_ at 37°C. The medium was changed three times a week. The Eca109/ABCG2 cells were maintained in RPMI-1640 supplemented with 10% FBS and 300 mg/l G418.

### Cytotoxicity assay

The sensitivity of the Eca109, Eca109/ABCG2 and Eca109/PCDNA3.1 cells to the anticancer drugs [adriamycin (ADM), daunorubicin (DNR) and mitoxantrone (MIT)] was determined using the 3-[4,5-dimethylthiazol-2-yl]-2,5-diphenyl tetrazolium bromide (MTT) assay, which is based on the capacity of viable cells to metabolize a yellow tetrazolium salt, MTT, using mitochondrial succinate dehydrogenase, into purple formazan crystals when dissolved in acidified propan-2-ol; the resulting purple solution is then spectrophotometrically measured at 490 nm. The cells were seeded into 96-well culture plates at a density of 5×10^4^ cells/ml. The serial concentrations of the anticancer drugs, ADM, DNR and MIT, were added in a final volume of 200 μl/well. Following the drug treatment for 72 h, the medium was replaced with an equal volume of fresh medium containing 0.5 mg/ml MTT and incubated for 4 h. The medium was removed and 180 μl DMSO was added and incubated for 10 min at room temperature. The cytotoxic effects of the drugs were determined according to the optical density (OD) values using a microplate reader at an absorption wavelength of 490 nm. The cell viability is expressed as the relative formazan formation in the treated samples compared with the control cells (A490 treated cells / A490 control cells × 100%).

### Flow cytometry

The level of ABCG2 protein expression, the apoptosis rate and the ADM content were analyzed in the Eca109 cells using flow cytometry.

The cells were harvested with trypsin-EDTA (1:20), washed with phosphate-buffered saline (PBS) and centrifuged for 5 min at 1,200 × g. The FITC-conjugated anti-ABCG2 antibody was added and the cells were incubated at room temperature for 30 min in the dark. The labeled cells were washed in PBS, centrifuged for 5 min at 1,200 × g and kept at 4°C until they were used.

The cells were dyed in 1 ml fluorescence liquor containing 50 μg/ml propidium iodide (PI) following incubation for 30 min in the dark at 4°C, then kept at 4°C until they were used.

The ADM in the Eca109 cells exhibited red fluorescence, which was detected by a 488 nm laser and analyzed using flow cytometry.

The cells were analyzed using a Beckman Coulter Epics-XL II type cytometer equipped with a 488 nm argon ion laser (Beckman Coulter, Miami, FL, USA). For each sample, 10,000 events selected in the living cell gate were measured. Forward scatter (FSC) and side scatter (SSC) data were used to establish a gate excluding the dead cells and debris.

### ADM accumulation and efflux by flow cytometry

The cellular accumulation and efflux of ADM were analyzed by flow cytometry. The Eca109, Eca109/PCDNA3.1 and Eca109/ABCG2 cells (4×10^5^) were incubated with 0.02 μg/ml ADM at 37°C for 2 h and washed twice with ice-cold PBS. The cells were resuspended in ADM-free RPMI-1640 for 1 h. The cells were washed with ice-cold PBS and the ADM that was retained in the cells was detected by flow cytometry.

In the drug efflux studies, 4×10^5^ Eca109/ABCG2 cells were incubated with 0.02 μg/ml ADM at 37°C for 2 h and washed twice with ice-cold PBS. The cells were resuspended in ADM-free RPMI-1640 in the absence or presence of 1 μmol/l Art for 1 h. The cells were washed with ice-cold PBS and the ADM that was retained in the cells was detected by flow cytometry.

### Total mRNA isolation and RT-PCR

Total RNA was extracted using TRIzol according to the manufacturer’s instructions and then treated with AMV reverse transcriptase to form cDNA (Promega Corporation, Madison, WI, USA). The cDNA was amplified by PCR using TaqDNA polymerase, which was performed by denaturation at 94°C for 30 sec, annealing at 57°C for 30 sec and extension at 72°C for 30 sec. This was performed for 35 cycles. The PCR-amplified products were run on a 1.5% agarose gel and visualized by ethidium bromide staining. The expression intensities of the optimized bands were quantified using Quantity One software (Bio-Rad, Mississauga, ON, Canada) and expressed as a ratio [ABCG2 vs. glyceraldehyde 3-phosphate dehydrogenase (GAPDH)]. The PCR primers were as follows: ABCG2 forward, 5′-GGT CAG AGT GTG GTT TCT GTA GCA-3′ and reverse, 5′-GTG AGA GAT CGA TGC CCT GCT TTA-3′ (product, 280 bp); and GAPDH forward, 5′-ACC ACA GTC CAT GCC ATC AC-3′ and reverse, 5′-TCC ACC ACC CTG TTG CTG TA-3′ (product, 452 bp).

### Western blotting

The ABCG2 protein expression level was detected by western blotting. Each cell line was grown to confluence, trypsinized, transferred to eppendorf tubes and rinsed with ice-cold PBS. The contents of each tube were suspended in 200 μl lysis buffer in the presence of protease inhibitors. The cell suspension was incubated for 20 min at 4°C and centrifuged for 10 min at 32,000 × g to obtain a clear supernatant. The protein concentration was measured by the Bio-Rad protein assay with bovine serum albumin (BSA) as a standard (Bio-Rad Laboratories, Mississauga, ON, Canada). For the western blot analysis, the protein extract was analyzed using SDS gel electrophoresis on appropriate polyacrylamide gels (5%) with 50 μg protein loaded on each lane. The proteins on the gels were then transferred to polyvinylidene difluoride (PVDF) membranes by a semi-dry transfer method. Subsequent to blocking with Tris-buffered saline (TBS) containing 5% skimmed milk and 0.1% Tween-20, the membranes were probed with a primary antibody against ABCG2 (mouse monoclonal antibody, clone no. sc-18841; Santa Cruz Biotechnology, Inc., Santa Cruz, CA, USA). The antibody was used at a dilution of 1:1,000. The membrane blots were then reacted with a secondary antibody (horseradish peroxidase anti-mouse immunoglobulin G), washed extensively with TBS (0.1% Tween-20) and submerged in 3,3′-diaminobenzidine (DAB), then developed to visualize the antibody-antigen complexes.

### Statistical analysis

The statistical analysis was performed using SPSS 11.5 software (SPSS, Inc., Chicago, IL, USA). The data are presented as the mean ± SD and three individual experiments were performed in triplicate. A one-way ANOVA and an LSD test were used to compare the data between three or more groups. P<0.05 was considered to indicate a statistically significant difference.

## Results

### Establishment of Eca109/ABCG2 cell line

**Figure 1:**
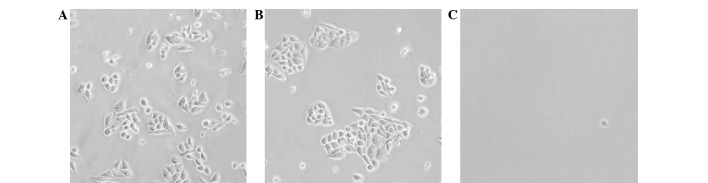
Morphological changes in the Eca109/ABCG2, Eca109/PCDNA3.1 and Eca109 cells following treatment with 600 mg/l G418 for 14 days, as observed by optical microscope (x100). Following the 600 mg/l G418 treatment for 14 days in the Eca109 cells transfected with PCDNA3.1-ABCG2 and PCDNA3.1, the positive clones were alive and the negative clones died. Therefore, the stably-transfected cells were selected by G418. (A) Eca109/ABCG2, (B) Eca109/PCDNA3.1 and (C) Eca109 cells. ABC, ATP-binding cassette.

The Eca109 cells that were transfected with PCDNA3.1-ABCG2 plasmids were selected by G418 for 14 days. The positive clones, i.e. the Eca109/ABCG2 cells, survived, but the negative clones died (Fig. 1). The Eca109 cells that were transfected with PCDNA3.1, i.e. the Eca109/PCDNA3.1 cells, were used as the control cells.

**Figure 2:**
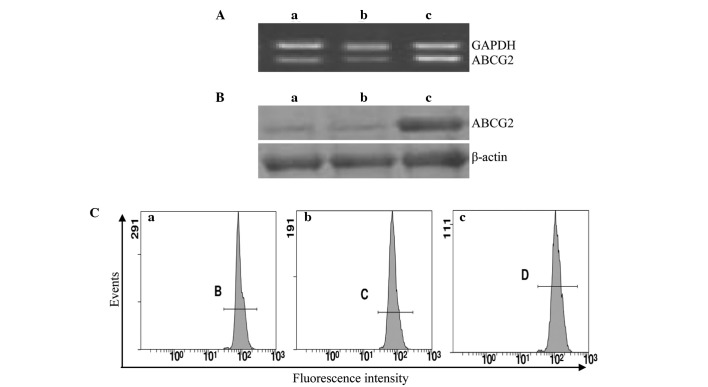
High expression level of ABCG2 in the Eca109/ABCG2 cells. (A) ABCG2 mRNA expression in the Eca109, Eca109/PCDNA3.1 and Eca109/ABCG2 cells was assayed by RT-PCR. GAPDH was used as a loading control. The ABCG2 mRNA expression level was higher in the Eca109/ABCG2 cells than that in the Eca109 and Eca109/PCDNA3.1 cells. (B) The ABCG2 protein level in the Eca109, Eca109/PCDNA3.1 and Eca109/ABCG2 cells was assayed by western blotting. The ABCG2 protein level in the Eca109/ABCG2 cells was higher than that in the Eca109 and Eca109/PCDNA3.1 cells. β-actin was used as a loading control. (C) The ABCG2 protein levels in the Eca109, Eca109/PCDNA3.1 and Eca109/ABCG2 cells were assayed by flow cytometry. The mean fluorescence intensity of the ABCG2 protein in the Eca109/ABCG2 cells was higher than that in the Eca109 and Eca109/PCDNA3.1 cells. The ABCG2 protein expression level was represented by the mean fluorescence intensity. (a) Eca109, (b) Eca109/PCDNA3.1 and (c) Eca109/ABCG2 cells. ABC, ATP-binding cassette; GAPDH, glyceraldehyde 3-phosphate dehydrogenase.

The expression of ABCG2 in the Eca109, Eca109/ABCG2 and Eca109/PCDNA3.1 cells was examined using RT-PCR, western blot analysis and flow cytometry. The ABCG2 expression level in the Eca109/ABCG2 cells was higher than that in the Eca109 and Eca109/PCDNA3.1 cells (P<0.05; Fig. 2; Table I).

### Resistance of Eca109/ABCG2 cells to anticancer agents

Following the anticancer drug treatment using ADM, DNR and MIT for 72 h, the IC_50_ was detected by MTT assay. The Eca109/ABCG2 cells were resistant to ADM, DNR and MIT compared with the Eca109 and Eca109/PCDNA3.1 cells, and the resistance to ADM was similar to the resistance to DNR and MIT (Table II).

### ADM efflux effect of Eca109/ABCG2 cells assayed by flow cytometry

**Figure 3:**
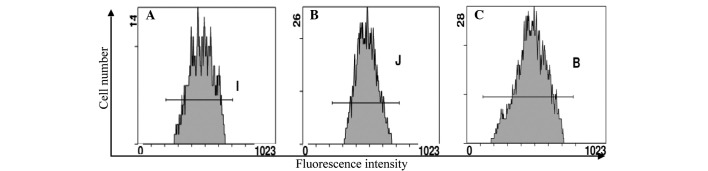
ADM efflux effect of the Eca109/ABCG2 cells assayed by flow cytometry. The Eca109, Eca109/PCDNA3.1 and Eca109/ABCG2 cells were incubated at 37°C with 0.02 μg/ml ADM for 2 h and then without ADM for 1 h. The ADM fluorescence intensity, which represents the ADM content in the cells, was detected by flow cytometry. The ADM fluorescence intensity in the Eca109/ABCG2 cells was lower than in the Eca109 and Eca109/PCDNA3.1 cells. The ADM efflux effect of the Eca109/ABCG2 cells was more efficient than that of the Eca109 and Eca109/PCDNA3.1 cells. (A) Eca109, (B) Eca109/PCDNA3.1 and (C) Eca109/ABCG2 cells. ABC, ATP-binding cassette; ADM. adriamycin.

To investigate how the Eca109/ABCG2 cells resisted the anticancer agents, the ADM efflux effect was investigated using flow cytometry (Fig. 3). The cells were incubated at 37°C with 0.02 μg/ml ADM for 2 h and then without ADM for 1 h. The level of ADM decreased more in the Eca109/ABCG2 cells than in the Eca109 and Eca109/PCDNA3.1 cells. The ADM efflux effect of the Eca109/ABCG2 cells was more than that of the Eca109 and Eca109/PCDNA3.1 cells.

### Reversal of drug resistance by Art in Eca109/ABCG2 cells

The ability of Art to reverse the drug resistance of the Eca109/ABCG2 cells was examined using an MTT assay (Table III). Art alone at 0.01, 0.1 and 1 μmol/l had no cytotoxic effects on the Eca109, Eca109/PCDNA3.1 and Eca109/ABCG2 cells. Therefore, 0.01, 0.1 and 1 μmol/l Art was used in this experiment. Art at 0.01 and 0.1 μmol/l moderately reversed the resistance of the Eca109/ABCG2 cells to ADM, while Art at 1 μmol/l completely reversed the resistance of the Eca109/ABCG2 cells to ADM.

### Effect of Art on the apoptosis of Eca109/ABCG2 cells induced by ADM

**Figure 4:**
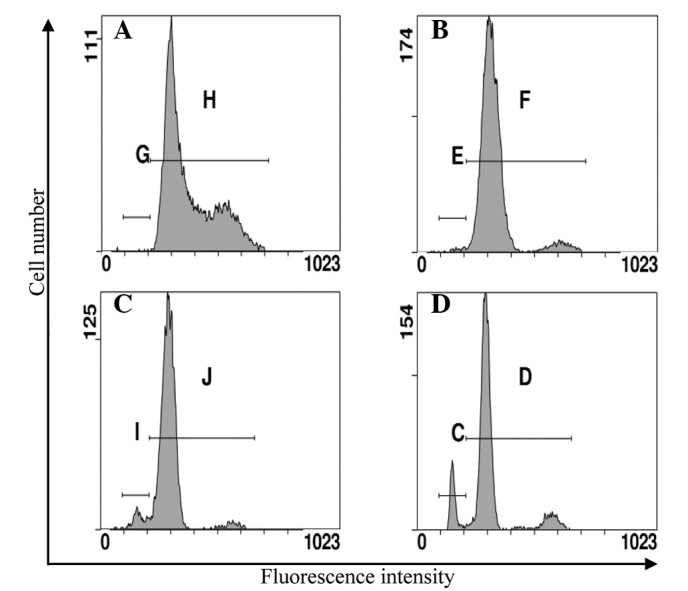
Apoptosis rate of the Eca109/ABCG2 cells following various drug treatments for 48 h, as detected by FCM. The cell apoptosis rate was higher in the 1 μmol/l Art + 0.2 mg/l ADM group than that in the 0.2 mg/l ADM, 1 μmol/l Art and NS groups. (A) NS, (B) 1 μmol/l Art, (C) 0.2 mg/l ADM and (D) 1 μmol/l Art + 0.2 mg/l ADM groups. ADM, adriamycin; Art, artesunate; ABC, ATP-binding cassette; FCM, flow cytometry.

To investigate the reversal of the drug resistance caused by Art in the Eca109/ABCG2 cells, the effect of Art on the apoptosis of the Eca109/ABCG2 cells induced by ADM was investigated. The apoptosis rate of the Eca109/ABCG2 cells following the treatment with ADM and Art was higher than that following the treatment with ADM alone (P<0.05; Fig. 4; Table IV).

### Effect of Art on the cellular accumulation and efflux of ADM

**Figure 5:**
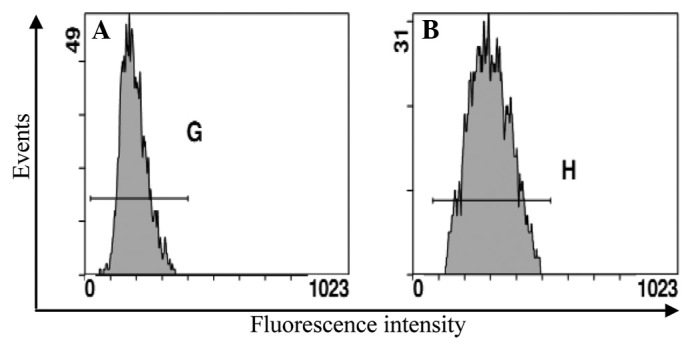
Effects of MDR-reversal agents on the accumulation and efflux of adriamycin (ADM) in the Eca109/ABCG2 cells. ADM was retained in the Eca109/ABCG2 cells in the absence or presence of 1 μmol/l Art following 2 h of ADM accumulation. The ADM content of the ADM + Art group in the Eca109/ABCG2 cells was higher than that of the ADM group. Art inhibited the efflux of ADM from the Eca109/ABCG2 cells. (A) ADM and (B) ADM + Art groups. MDR, multidrug resistance; ABC, ATP-binding cassette; Art, artesunate.

To investigate how Art reversed the resistance of the Eca109/ABCG2 cells to the anticancer agents, the effect on the accumulation of ADM was investigated using flow cytometry. The increased accumulation of ADM in the Eca109/ABCG2 cells caused by Art was due to the inhibition of the ADM efflux (Fig. 5). When the cells were incubated at 37°C with 0.02 μg/ml ADM for 2 h and then without ADM and with or without 1 μmol/l Art for 1 h, the level of ADM in the Eca109/ABCG2 cells with Art showed a smaller decrease than that without Art. Art inhibited the efflux of ADM from the Eca109/ABCG2 cells.

### Effect of Art on the expression level of ABCG2 in Eca109/ABCG2 cells

**Figure 6:**
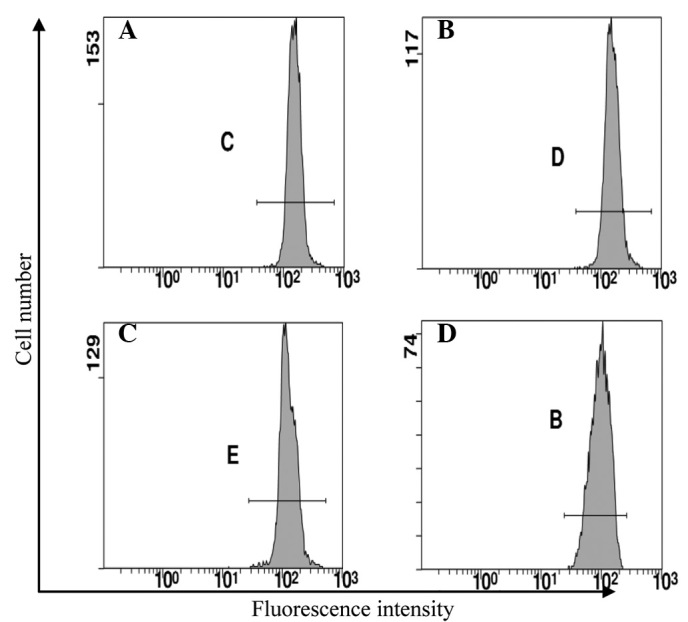
Effect of Art on the expression level of ABCG2 in the Eca109/ABCG2 cells, as detected by flow cytometry. Art downregulated the expression level of the ABCG2 protein in a concentration-dependent manner. (A) 0 μmol/l Art; (B) 0.01 μmol/l Art; (C) 0.1 μmol/l Art; and (D) 1 μmol/l Art. ABC, ATP-binding cassette. Art, artesunate.

To investigate the mechanism of the reversal of resistance caused by Art in the Eca109/ABCG2 cells the effect of Art on the expression level of ABCG2 in the Eca109/ABCG2 cells was investigated. Art inhibited the expression level of ABCG2 protein in the Eca109/ABCG2 cells (Fig. 6).

## Discussion

Chemotherapy is the most common treatment for esophageal carcinoma, particularly for advanced esophageal cancer and recurrent cancer. However, the response from patients with recurrent esophageal cancer remains poor. Drug resistance is major obstacle for chemotherapy results and is attributable to several processes that occur in neoplastic cells. One of these processes is the decreased accumulation of anticancer drugs within the cancer cells due to drug efflux, which is mediated by ABC transporters (19,20). Overexpression of these transporters results in drug resistance in a number of tumors (21,22). However, the significance of the expression of the ABC protein in esophageal cancer has not yet been reported. The present study investigated a potential correlation between ABCG2 expression and MDR in esophageal cancer.

ABCG2 is a member of the ABC family and functions as a ABC discharge pump. ABCG2 was initially isolated from a doxorubicin-resistant MCF-7/AdrVP breast cancer cell line (21). ABCG2 is a 655-amino acid, 72-kDa protein and the gene is located on chromosomal locus 4q22. The majority of ABC transporters, including P-gp and MRPs, have two ATP-binding domains and two sets of transmembrane domains (7). In contrast, ABCG2 has a single ATP-binding domain at the amino terminus and a single set of transmembrane domains at the carboxyl terminus. Thus, ABCG2 is a half ABC transporter. Previous studies revealed that ABCG2 is expressed in a variety of tumor cells and human solid tumors (9,22). ABCG2 was demonstrated to be expressed in numerous normal human tissues (23,24), including placental syncytiotrophoblasts, brain blood capillaries, testes, small intestine, colon, kidneys and liver. Among these tissues, ABCG2 has been identified to be localized in the apical border of a variety of secretory epithelia, indicating its role in the protection of the cells against exogenous toxins, including anticancer agents (23,24). ABCG2 prevents the intracellular accumulation of substrate compounds, including anticancer drugs, by limiting the influx into and facilitating the efflux out of cells. In normal tissues, ABCG2 may play a significant role in the protection of the fetus or organism from toxic xenobiotics (23). In cancer cells, a high expression of ABCG2 prevents the intracellular accumulation of anticancer drugs, which results in drug resistance (21). The present study discussed the association between ABCG2 expression and MDR in esophageal cancer.

In order to investigate the correlation between drug resistance in esophageal cancer and ABCG2 expression, the ABCG2 gene was transfected into the Eca109 esophageal cancer cell line to establish Eca109/ABCG2 cells. The expression level of ABCG2 in the Eca109/ABCG2 cells was higher than that in the Eca109 and Eca109/PCDNA3.1 cells (Fig. 2). The Eca109/ABCG2 cells showed cross-resistance to ADM, DNR and MIT compared with the Eca109 and Eca109/PCDNA3.1 cells (Table I). The drug efflux effect of the Eca109/ABCG2 cells was stronger than that of the Eca109 and Eca109/PCDNA3.1 cells (Fig. 3). The Eca109/ABCG2 cell line was multidrug resistant. A high expression of ABCG2 in the cells enhanced the drug efflux effect of ADM, DNR and MIT. Therefore increasing the expression level of ABCG2 resulted in MDR.

Resistance reversal agents for the ABC protein have been studied and due to the greater toxicity, few agents are used in clinical applications. The identification of a reversal agent with low toxicity and efficient resistance is required. Art is a derivative of artemisinin. Studies have identified that artemisinin and its derivatives are anti-inflammatory, antifibrosis, fight schistosomiasis and antitumor agents. Art is a good antimalarial drug, particularly for heavy and drug-resistant malaria, however, studies have paid more attention to the antitumor function of Art (13–16). The antitumor effect of Art has not produced cross resistance (17), indicating that Art confers a reversal to drug resistance. The present study investigated the function and mechanism of Art in the reversal of esophageal cancer drug resistance. The esophageal cancer drug resistant Eca109/ABCG2 cell line was established by transfecting the ABCG2 gene into Eca109 cells. The Eca109/ABCG2 cells had a high expression of the ABCG2 gene and protein compared with the Eca109 cells (Fig. 2). High ABCG2 expression in the cells resulted in MDR. The Eca109/ABCG2 cells were resistant to ADM, DNR and MIT (Table I). The mechanism of MDR that was induced by ABCG2 was detected for further investigation. The ADM content in the Eca109/ABCG2, Eca109/PCDNA3.1 and Eca109 cells was identified using flow cytometry following the treatment with ADM. The ADM content in the Eca109/ABCG2 cells was lower than in the Eca109/PCDNA3.1 and Eca109 cells (Fig. 3), indicating that the drug efflux effect of Eca109/ABCG2 was stronger than that of the other two types of cells. The Eca109/ABCG2 cells were a cell line with an ABCG2 drug-resistant phenotype. ABCG2-mediated drug secretion reduced the level of anticancer drugs in the Eca109/ABCG2 cells, which caused MDR.

The inhibitory effect of ADM on the Eca109/ABCG2 cells was enhanced by combining the drug with Art (Table II). The apoptosis rate of the Eca109/ABCG2 cells that was induced by ADM was also enhanced by combining the drug with Art (Fig. 4). These results suggested that Art had a role in the reversal of drug resistance. For the next level of investigation, the mechanism behind the reversal of drug resistance by Art was investigated. Art reduced the level of ABCG2 expression (Fig. 6) and inhibited the drug efflux effect (Fig. 5) of the Eca109/ABCG2 cells. Art was able to reverse drug resistance by reducing ABCG2 expression and increasing the anticancer drug concentration in the cancer cells.

In summary, ABCG2 expression participated in the MDR of esophageal cancer. Art was able to reverse the drug resistance by reducing ABCG2 expression and increasing the anticancer drug concentration in the cancer cells. Art is expected to be associated with low toxic side-effects and may be a highly efficient resistance reversal agent in the clinic.

## Figures and Tables

**Table I:** Expression level of ABCG2 in various cells detected by RT-PCR, western blot analysis and FCM.

Cell	ABCG2 mRNA^a^	ABCG2 protein^b^	ABCG2 protein^c^
Eca109	0.68±0.04^d^	0.33±0.04^d^	628.25±12.49^d^
Eca109/PCDNA3.1	0.67±0.04^d^	0.36±0.05^d^	627.59±9.62^d^
Eca109/ABCG2	0.93±0.05	0.74±0.05	689.93±10.15

a ABCG2 mRNA was detected by RT-PCR assay.

b ABCG2 protein was detected by western blot assay.

c ABCG2 protein was detected by FCM assay. Values are presented as the mean ± SD of six experiments.

d P<0.05 vs. Eca109/ABCG2 cell group.

ABC, ATP-binding cassette; FCM, flow cytometry.

**Table II:** Resistance of Eca109/ABCG2 cells to anticancer agents.

	IC_50_		
	
Drug	Eca109	Eca109/PCDNA3.1	Eca109/ABCG2
ADM	1.14±0.06 (1.00)^a^	1.16±0.06 (1.02)^a^	6.80±0.07 (5.96)
DNR	0.41±0.07 (1.00)^a^	0.39±0.07 (0.95)^a^	2.32±0.07 (5.66)
MIT	0.49±0.06 (1.00)^a^	0.47±0.06 (0.96)^a^	2.71±0.06 (5.53)

Cell survival was determined by MTT assay. Values are presented as the mean ± SD of nine experiments (mg/l). Relative resistance (values in brackets) was determined by dividing the IC_50_ values of the drugs for the Eca109/PCDNA3.1 and Eca109/ABCG2 cells by that for the Eca109 cells.

a P<0.05 vs. Eca109/ABCG2 cell group.

ADM, adriamycin; DNR, daunorubicin; MIT, mitoxantrone; MTT, 3-[4,5-dimethylthiazol-2-yl]-2,5-diphenyl tetrazolium bromide.

**Table III:** Effects of Art on the cytotoxicity of ADM for the Eca109, Eca109/PCDNA3.1 and Eca109/ABCG2 cells.

	IC_50_		
	
Drug	Eca109	Eca109/PCDNA3.1	Eca109/ABCG2
ADM	1.14±0.06 (1.00)	1.16±0.06 (1.02)	6.80±0.07 (5.96)
+Art (0.01 μmol/l)	1.13±0.07 (1.00)	1.14±0.06 (1.01)	4.64±0.07 (4.11)^a^
+Art (0.1 μmol/l)	1.10±0.06 (1.00)	1.13±0.07 (1.03)	2.45±0.06 (2.23)^a^
+Art (1 μmol/l)	1.08±0.07 (1.00)	1.11±0.07 (1.03)	1.21±0.06 (1.12)^a^

Cell survival was determined by MTT assay. Values are presented as the mean ± SD of nine experiments (mg/l). Relative resistance (values in brackets) was determined by dividing the IC_50_ values of ADM for Eca109/PCDNA3.1 and Eca109/ABCG2 cells with or without Art by that for Eca109 cells with or without Art.

a P<0.05 vs. ADM group.

ADM, adriamycin; Art, artesunate; ABC, ATP-binding cassette ; MTT, 3-[4,5-dimethylthiazol-2-yl]-2,5-diphenyl tetrazolium bromide.

**Table IV:** Apoptosis rate of Eca109/ABCG2 cells following various drug treatments for various times, as detected by FCM.

	Apoptosis rate, %		
	
Drug	24 h	48 h	72 h
ADM (0 mg/l)	1.29±0.60	1.17±0.33	1.28±0.31
ADM (0.2 mg/l)	3.78±0.13	7.22±0.35	9.94±0.08
+Art (0.01 μmol/l)	4.18±0.27	8.69±0.50	13.43±0.52
+Art (0.1 μmol/l)	7.18±0.32^a^	13.01±0.46^a^	17.81±0.24^a^
+Art (1 μmol/l)	9.99±0.29^a^	17.15±0.89^a^	22.95±0.08^a^

Values are presented as the mean ± SD of six experiments (%).

a P<0.05 vs. ADM (0.2 mg/l) group.

ADM, adriamycin; Art, artesunate; ABC, ATP-binding cassette; FCM, flow cytometry.
